# Top 100 most-cited articles on pelvic organ prolapse: a visualization and bibliometric analysis

**DOI:** 10.3389/fsurg.2025.1485426

**Published:** 2025-03-28

**Authors:** Lu Yang, Qin Tao, Huaye Wu, Litong Yin, Xuemei Wu, Ying Xiong, Xiaoqin Gan, Yonghong Lin, Xia Yu

**Affiliations:** ^1^ Chengdu Women’s and Children’s Central Hospital, School of Medicine, University of Electronic Science and Technology of China, Chengdu, China; ^2^ Department of Clinical Laboratory, Chengdu Women’s and Children’s Central Hospital, Sichuan Provincial People’s Hospital, School of Medicine, University of Electronic Science and Technology of China, Chengdu, Sichuan, China

**Keywords:** bibliometric, pelvic organ prolapse, research directions, visualization, CiteSpace

## Abstract

**Introduction:**

Bibliometric analysis is a scientometric method that allows the quantitative analysis of publications. This study used the Web of Science database to perform a bibliometric analysis of the 100 most-cited articles in the field of pelvic organ prolapse (POP) to identify the key research themes and emerging topics within this area.

**Methods:**

We collected pertinent publications from the Web of Science Core Collection (WoSCC). The search was conducted using the following keywords: “pelvic organ prolapse”, “pelvic organ prolapse” or “pelvic organ prolapses”. The search included all publication dates up to June 27, 2024, without any article type restrictions, and the articles were sorted based on their citation count. The top 100 articles with the most citations were included in the subsequent analyses. Several tools, including the Bibliometrix program in R, CiteSpace software, the Online Analysis Platform of Literature Metrology (https://bibliometric.com), and an online interface by Bibliometrix, were used to analyze the data.

**Results:**

The top 100 most cited articles in the field of POP were cited 26,894 times in total. These articles were published between 1993 and 2019, and the majority of them were published during the 10-year period from 2001 to 2010. The United States and the University of California, San Francisco, produced the most publications on this topic. The American Journal of Obstetrics and Gynecology had the greatest influence on POP research. The most prolific author in this field was Barber MD (*n* = 7). Epidemiological research and treatment, particularly in the area of tissue engineering, were the main focus and current trends in POP research.

**Conclusion:**

This study revealed key research areas and current research hotspots for POP, with a particular focus on epidemiological studies and surgical interventions, especially in the field of tissue engineering. It is suggested that the future research in this field should pay more attention to epidemiological research and treatment, so as to better understand the risk factors of the disease and the characteristics of the affected population, and expect high-quality curative effect and prevention.

## Introduction

Pelvic organ prolapse (POP) occurs when pelvic organs descend into or outside the vagina, leading to symptoms such as vaginal bulging, pelvic pressure, urinary incontinence, and pelvic or low back pain (1). The prevalence of POP varies from 1.9% to 46.50% globally (2–4), significantly impacting women's health and quality of life (5–7).

Research indicates a higher incidence of prolapse surgery among older individuals (8). With the aging population, the prevalence of prolapse in women is projected to increase to 4.9 million by 2050, which represents a 46% increase and poses a significant societal burden (9). Hence, it is important to review the relevant literature to understand current research trends and key areas of interest in POP.

**Figure 1:**
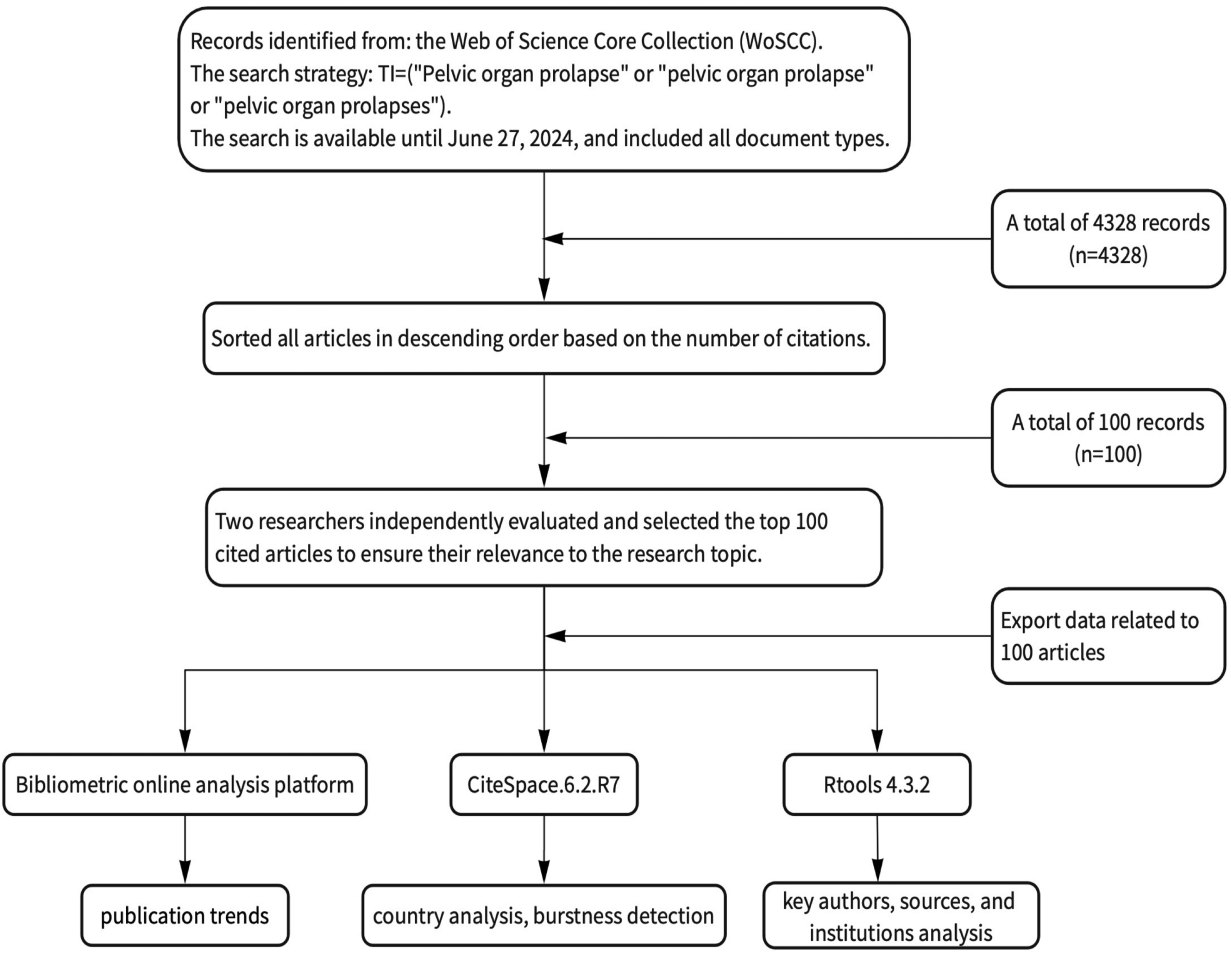
The working flow chart.

Bibliometric analysis is a scientific research tool that can quantitatively analyze publications and is used extensively for processing large datasets. It enables the examination of research trends, productivity, scientific linkage patterns, and the impact of scientific literature across various fields (10–12). This tool is widely applied in the medical domain, with software such as CiteSpace to facilitate the visualization of bibliometric results (13–15). One method employed in this analysis assesses how frequently an article is cited by other researchers. The number of citations of an article is a crucial indicator of its influence, it not only reflects the research status in this field, but also provides an important reference and foundation for future research (16–18). Therefore, the articles with the highest citation rates are likely to have the most significant impact within their scientific community. In addition, we found that previous studies in different research fields conducted data analysis by including the top 100 most-cited articles, so we also included the top 100 most-cited articles in the analysis. The working flow chart of this study is shown in Figure 1.

While bibliometric analysis has been applied to study trends in female pelvic organ research (19), no bibliometric analysis has been conducted to reveal the research emphasis and trajectory on highly cited articles in POP. To fill this gap, we conducted a bibliometric analysis of the most influential studies in the field of POP.

## Materials and methods

### Data sources and search strategies

Relevant articles were identified by searching the Social Sciences Citation Index (SSCI) and Science Citation Index Expanded (SCI-Expanded) on the Web of Science Core Collection (WoSCC) to mitigate potential bias resulting from database updates. The following search strategy was used: TI = (“pelvic organ prolapse” or “pelvic organ prolapse” or “pelvic organ prolapse”). The search included all publication dates up to June 27, 2024 and all study types. A total of 4,328 articles were retrieved from the WoSCC database. These articles were then sorted in descending order based on the number of citations, and the top 100 most cited articles were selected for further analysis. Initially, the titles of the articles were screened, and the abstracts and full texts were reviewed as needed. Two researchers (LY and XY) independently evaluated each article to ensure its relevance to the study topic.

In the field of epidemiology, articles were sorted into specific groups according to the type of research conducted: (1) experimental study; (2) review; (3) meta-analysis; (4) guidelines/consensus; and (5) observational study (prospective analyses, retrospective analyses and cross-sectional studies). The impact factor (IF) of the journals was extracted from Journal Citation Reports (JCR), and tables and graphs were generated using Microsoft Word and Excel, respectively. The study involved the analysis of published articles, thereby obviating the requirement for ethical clearance.

### Data analysis and data visualization

The data, sourced as a tab-separated text document from WoSCC, were imported into Bibliometrics' Online Analysis Platform for further analysis. The publication trends over the years were examined using the “Total volume” option.

The complete records and reference sources of the top 100 most cited articles were acquired in.TXT format from the WoSCC database. The identification of popular search terms was found based on burst points, while the countries of publication were obtained through a country analysis using specific parameters in the CiteSpace program.

Utilizing the Bibliometrix package in R (Version 4.3.2), we retrieved the top 100 articles, their associated citation counts and annual citations, and identified key authors, sources, and institutions from data stored in TXT format through the “Biblioshiny” web interface.

### Quality control of bibliometric analysis

We took the following quality control measures: (1) Data cleaning: We removed redundant and erroneous data and ensured that all data were in a consistent format to improve the accuracy of the analysis. (2) Tool calibration: We used the latest version of the bibliometric tool and calibrated the output results by comparing them with existing standards and datasets. (3) Sample representativeness: When selecting literature data, we ensured that sufficient sample time periods were included to maintain the representativeness of the results. (4) Result verification: Our results have been cross-checked via multiple independent analyses to ensure reliability. In addition, we invited experts in the field to review our results to ensure validity. (5) Transparency: We have documented the methodological process in detail so that future researchers can reproduce the study.

## Results

### The top 100 most cited articles and the number of citations

Supplementary Material 1 provides a list of the top 100 articles with their citation counts and average citations per year. The total number of citations for these articles was 26,894, with individual citations ranging from 98 to 3,255, an average of 268.94 citations per article and a median of 162. The average was calculated to better reflect the overall average of the data. The median represents the middlemost data point. Compared with the average, the median is not affected by extreme values and better reflects the central tendency of the data.

### Study types

**Figure 2:**
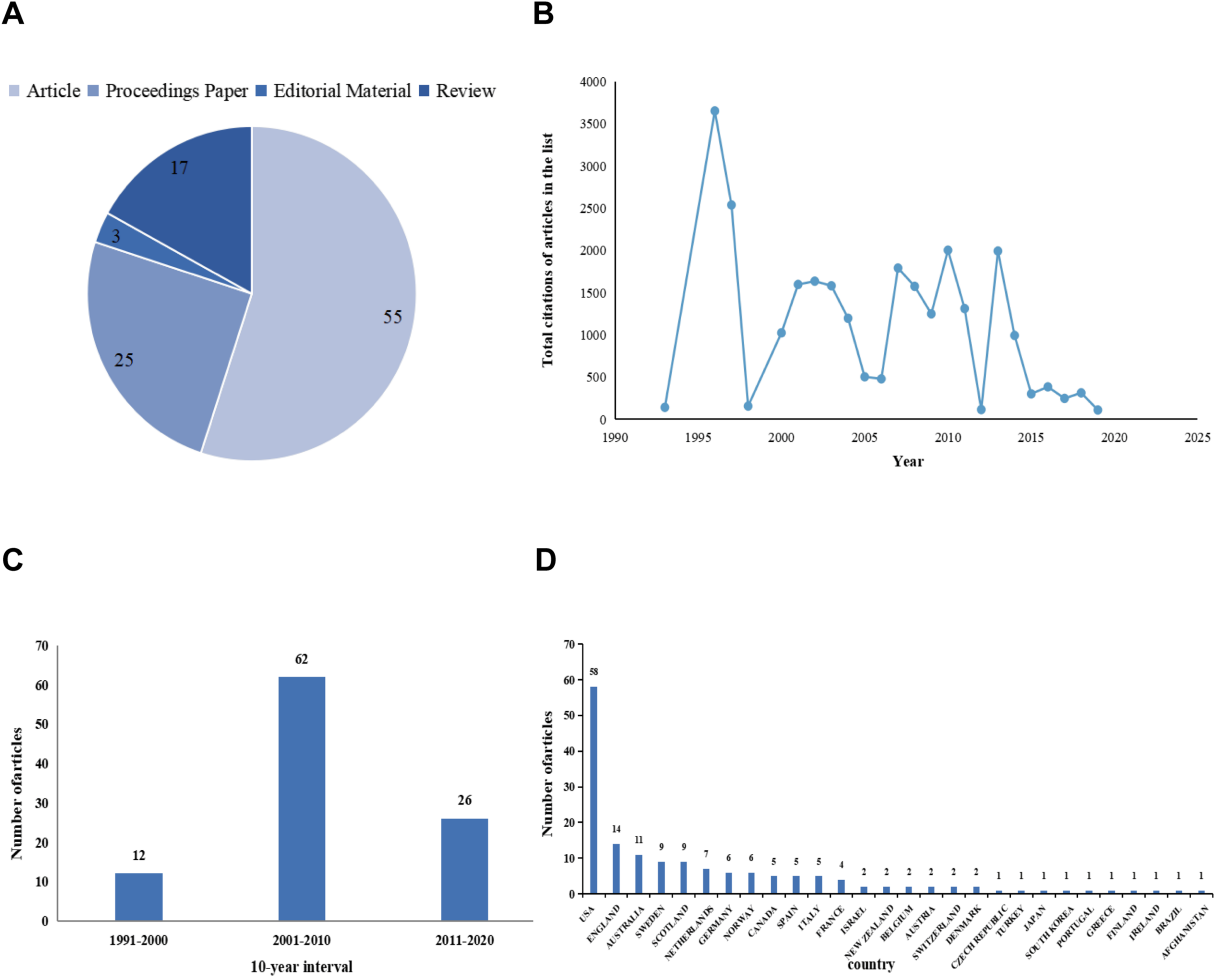
**(A)** Document types pie chart; **(B)** total number of citations per year; **(C)** annual publication volume at 10-year intervals; **(D)** number of publications by country.

Among the 100 articles analyzed, 55 were classified as original articles, 25 as proceedings papers, 17 as reviews, and 3 as editorial content (Figure 2A). The article with the highest number of citations, “The standardization of terminology of female pelvic organ prolapse and pelvic floor dysfunction,” was published in the American Journal of Obstetrics and Gynecology in 1996 and had 3,255 citations. This seminal work contributed significantly to the development of a standardized lexicon for characterizing POP and pelvic floor dysfunction in the female population.

### Total number of citations per year

Figure 2B represents the cumulative citations for the top 100 articles annually from 1993 to 2019. We refer to the method of analyzing the distribution of published papers at a 10-year interval adopted by existing studies, and conclude that the peak in article publications occurred between 2001 and 2010, as shown in Figure 2C.

### Country analysis

After eliminating duplicate entries, 100 articles were analyzed using CiteSpace. The top-cited articles originated from 28 countries, predominantly the United States (*n* = 58), followed by England (*n* = 14) and Australia (*n* = 11). Together, these three countries accounted for 83% of the total publications. The distribution of article contributions by country is illustrated in Figure 2D.

### Institution analysis

Table 1 displays a summary of institutions that authored six or more articles in the field of POP. Prominent contributors included the University of California, San Francisco (*n* = 12), the University of Aberdeen (*n* = 11), and the University of Michigan (*n* = 10).

**Table 1:** Institutions affiliated with 6 or more articles in the list.

Institution	Number of articles
Univ Calif San Francisco	12
Univ Aberdeen	11
Univ Michigan	10
Univ New Mexico	9
Karolinska Inst	8
Univ Pittsburgh	8
Univ Texas	8
Brown Univ	7
Cleveland Clin FDN	7
Madigan Army Med CTR	7
Obstet Gynecol and Womens Hlth Inst	7
Univ Med CTR	7
Univ Texas Sw Med CTR Dallas	7
Glasgow Caledonian Univ	6
Johns Hopkins Univ	6
Med Univ S Carolina	6
Radboud Univ Nijmegen	6

### Journal analysis

The search results from the WoSCC revealed that the 100 most cited articles were published across 22 journals, as shown in Table 2. We organized these journals by volume and included information on their 2023 IFs, JCR categories, and countries of publication. These data, sourced from journal citation reports, are crucial for assessing a journal's influence within the scientific community and identifying current research trends. The American Journal of Obstetrics and Gynecology was identified as the most significant contributor to research on POP, with 28 articles on the list and a total of 8,961 citations, boasting an IF of 8.7 in 2023. Following closely behind was obstetrics and gynecology. Notably, the Lancet held the highest IF for 2023 among these journals, at 98.4.

**Table 2:** Journals with their impact factors and total number of articles in the list.

No.	Sources	Articles	Category quartile	2023 impact factor	Total no. of citations
1	American Journal of Obstetrics and Gynecology	28	Q1	8.7	8,961
2	Obstetrics and Gynecology	22	Q1	5.7	7,463
3	International Urogynecology Journal	17	Q3	1.8	3,026
4	BJOG-an International Journal of Obstetrics and Gynaecology	4	Q1	4.7	797
5	Neurourology and Urodynamics	4	Q3	1.8	1,228
6	Cochrane Database of Systematic Reviews	3	Q1	8.8	1,138
7	European Urology	3	Q1	25.3	364
8	International Urogynecology Journal and Pelvic Floor Dysfunction	3	Q3	1.8	805
9	Lancet	3	Q1	98.4	842
10	American Journal of Pathology	1	Q1	4.7	143
11	American Journal of Roentgenology	1	Q1	4.7	171
12	BJU International	1	Q1	3.7	110
13	BMJ Open	1	Q1	2.4	111
14	BMJ-British Medical Journal	1	Q1	93.6	111
15	European Journal of Obstetrics & Gynecology and Reproductive Biology	1	Q2	2.1	179
16	JAMA-Journal of the American Medical Association	1	Q1	63.1	381
17	Journal of Clinical Investigation	1	Q1	13.3	108
18	Journal of Sexual Medicine	1	Q1	3.3	102
19	Journal of the American Association of Gynecologic Laparoscopists	1	Q2	1.843	120
20	New England Journal of Medicine	1	Q1	96.2	432
21	Ultrasound in Obstetrics & Gynecology	1	Q1	6.1	187
22	World Journal of Urology	1	Q2	2.8	115

### Author analysis

Overall, 454 authors were included in the 100 articles. The top 13 authors in the field of POP, each with a minimum of 4 articles, are presented in Table 3. The leading group was Barber MD, with 7 articles, followed by Maher C, Nygaard I, and Weber AM, with 5 articles each. Notably, a strong positive correlation was observed among the top 13 authors between their H-index and the number of articles on the list.

**Table 3:** Authors with 4 or more articles in the list.

Authors	Articles	h_index	PY_start	TC
Barber MD	7	7	2000	2,110
Maher C	5	5	2007	2,167
Nygaard I	5	5	2002	2,595
Weber AM	5	5	1997	1,056
Altman D	4	4	2007	739
Brubaker L	4	4	2009	1,671
Bump RC	4	4	1993	3,820
Cundiff GW	4	4	1996	922
Hagen S	4	4	2010	746
Handa VL	4	4	1996	664
Rogers RG	4	4	2001	1,062
Subak LL	4	4	2001	916
Word RA	4	4	2002	481

### Clusters of keywords

The burstness detection by the CiteSpace can extract burst terms from a large number of subject words in the literature to identify the research frontier and development trend of a certain discipline. Through burst point detection using CiteSpace software, 62 prominent terms were identified. After redundant and vague terms were removed, the remaining citations were categorized into seven subgroups: treatment, outcomes, anatomy and symptoms, diagnosis and detection, pathogenesis and risk factors, implants, and research proceedings (Supplementary Material 2). Terms with greater strength are associated with more robust research fields. Further, the duration of the burst time directly correlates with the longevity of the epidemic in a given time.

In the treatment subcluster, there was a notable transition from conventional hysterectomy to diverse transvaginal or transabdominal repair surgeries (Supplementary Material 2a). Patient outcomes, including physical and psychological well-being and overall quality of life, showed relatively high levels of satisfaction, as indicated by the prominence of specific keywords (Supplementary Material 2b). Notable keywords in the anatomy and symptoms subclusters, such as endopelvic fascia plication and vaginal wall prolapse, along with floor disorders, demonstrated significant citation bursts, with the keyword “floor disorder” exhibiting a substantial burst factor of up to 2.57 between 2008 and 2013 (Supplementary Material 2c). Among these, the keyword “stress incontinence” had citation bursts over the past decade (Supplementary Material 2d). Within the pathogenesis and risk factors subcluster, keywords associated with citation bursts shifted from discussions on innervation to elastic fiber homeostasis, age, and childbirth (Supplementary Material 2e). Conversely, the implants subcluster was characterized by a single keyword related to the utilization of implants in pelvic repair surgery (Supplementary Material 2f). Lastly, in research proceedings, keywords such as “randomized trial” and “prospective randomized trial” have shown robust citation bursts (Supplementary Material 2g).

### Article classification analysis

Table 4 shows the classification of the articles based on their study type, which includes experimental studies, observational studies, reviews and meta-analyses, and guidelines/consensuses.

**Table 4:** Classification of articles based on study type.

Types of study	No of articles
Guidelines/consensus	7
Meta-analysis	3
Reviews	17
Observational study	56
Retrospective	21
Prospective	14
Cross-sectional study	21
Experimental study	17

Seventeen studies were experimental research conducted to identify the mechanisms underlying disease development and progression, compare treatment modalities, and monitor patient prognosis. Fifty-six articles were observational studies, employing prospective, retrospective, or cross-sectional designs. Twenty-one studies were cross-sectional, fourteen were prospective, and the remainder were retrospective studies. Experimental research aimed primarily to evaluate the efficacy and safety of treatments and explore personalized approaches. Additionally, seventeen articles were comprehensive reviews that addressed the pathogenesis of POP to treatment strategies for POP. Notably, only three articles were meta-analyses, providing evidence-based insights into nonsurgical and surgical interventions for POP. Furthermore, seven research articles outlined the fundamental principles, recommendations, and consensus reached by experts guiding clinical decision-making for healthcare professionals and caregivers.

A comprehensive examination of 33 research articles was performed to elucidate the key mechanisms underlying the onset of POP. Factors such as vaginal childbirth, forceps delivery, fetal weight, neuromuscular injury, racial disparities, obesity, changes in vaginal biomechanics, modifications in connective tissue structure and function, advancing age, increased body mass index, and other variables were investigated. The development of prolapse is influenced by multiple factors, with vaginal childbirth, advanced age, and increased body mass index emerging as the most prevalent risk factors.

Forty-six articles analyzed the treatment and prognosis of POP, assessed the safety and effectiveness of both surgical and nonsurgical interventions, as well as explored complications such as hysterectomy, abdominal sacrocolpopexy, vaginal sacrospinous colpopexy, posterior vaginal wall repair, mesh repair for anterior or posterior compartment prolapse, and pelvic floor muscle training. Moreover, reoperation rates were monitored for patients who underwent surgical treatment for POP. This condition impacts women throughout their reproductive years, prompting individuals of all ages to seek surgical intervention.

The research in three studies focused on imaging, specifically the use of ultrasonic diagnosis and dynamic MR imaging for the detection and measurement of POP. Furthermore, eighteen studies investigated symptoms and treatment, establishing standardized terminology and management strategies for patients with POP.

## Discussion

In the fields of obstetrics and gynecology, POP is a significant research subject. In this study, we analyzed 100 articles to provide a thorough examination of the most frequently cited research on POP. Through bibliometric analysis, our aim was to systematically identify key research trends and advancements. Additionally, by categorizing studies based on topics and article descriptors, we highlighted critical areas within POP that are currently under investigation and may require further research attention.

Although the study of POP dates back to 1975 (19), our analysis revealed that the majority of highly cited studies were conducted after 1993. The period from 1993 to 2019 had the highest number of top 100 cited articles, with peak research activity observed between 2001 and 2010. During this timeframe, a total of 62 articles were published, with notable contributions in 2008 and 2009, adding 10 and 7 articles, respectively, to the list. Our current research trends align with bibliometric analyses in related areas, including studies on female POP, trends in pelvic floor reconstruction, and POP repair using mesh (19–21).

It is a well-established phenomenon that newer articles, despite potentially having a greater impact, may rank lower based on citations. This discrepancy arises from the lower number of citations for newer articles in their initial years and their limited time frame to accumulate citations, consequently leading to reduced representation in the top 100 article list (22). Recognizing the limitations of relying solely on citations as a metric of scientific value, we adopted citations per year as a more robust parameter for evaluating an article's enduring impact. Nevertheless, our analysis revealed that, even when ranked by citations per year, the most cited articles consistently maintained the highest total citation counts.

Topics related to POP span various disciplines, including neurology, urology, basic science, and general medicine, leading to the publication of highly cited articles in a range of journals. While a small number of articles (*n* = 4) were found in neurology journals, the majority were published in obstetrics and gynecology journals. This publishing pattern mirrors bibliometric trends observed in other fields, such as thyroid eye disease and regenerative endodontics, where top articles are predominantly found in specialized journals due to the focus on advancements appealing primarily to researchers and practitioners within the specialty areas (23, 24).

The preponderance of publications emanated from nations such as the U.S. (*n* = 58), with notable contributions from England, Australia, Sweden, and Scotland. In a bibliometric examination of POP from 2007 to 2016, Australia was not listed among the top five high-producing countries (25). Nevertheless, given the present surge in publications, numerous countries beyond the U.S. have made substantial contributions to the academic literature in POP research.

The top 10 academic institutions that contribute the most are mainly from the U.S. and the UK. None of the institutions with over six publications were linked to a developing country, and this could be ascribed to the delayed or slower advancements in research development in those regions.

The list predominantly featured original research articles, with review articles constituting less than twenty percent. Only three meta-analyses were included. Close to half of the articles addressed POP treatment, while approximately one-third explored the pathogenesis and pathophysiology of POP. This distribution underscores the importance of epidemiological research and treatment in POP, indicating the continued investigation on POP pathogenesis.

In our analysis, we observed a shift in anatomical research within the field of POP, from focusing on structural aspects to emphasizing the pelvic floor ligament. This finding is consistent with previous studies that explored the pathogenesis of POP at the microscopic level of the pelvic ligament (26). In addition, interest in the use of polypropylene mesh has increased, suggesting that tissue engineering has become a new research hotspot. This is consistent with the results of previous studies (19, 27). Furthermore, current research on POP involves the investigation of various cell types, including stem cells, primary somatic cells, and specialized cell lines (28, 29). Additionally, there is ongoing investigation of bionic scaffolds such as absorbable poly-4-hydroxybutyrate (30).

Bibliometrics, as a quantitative measure, primarily reflects an article's recognition within the scientific community rather than its quality and potential impact on research. Despite employing a rigorous analytical approach, our focus was limited to the top 100 most cited articles, potentially overlooking other valuable literature. Additionally, the “cumulative effect” of earlier published articles, influenced by varying publication times and the fact that low citations do not necessarily signify poor research quality, should be considered. Furthermore, while bibliometric analysis aids in identifying influential research, crucial factors such as research design and experimental method rigor are not factored in. In conclusion, the outcomes of our bibliometric analysis can provide insights into monitoring the latest developments and popular research areas in the field of POP.

## Data Availability

The original contributions presented in the study are included in the article/Supplementary Material, further inquiries can be directed to the corresponding authors.
