# Supplementary material for: Top 100 most-cited articles on pelvic organ prolapse: a visualization and bibliometric analysis
Source: Front Surg. 2025 Mar 28;12:1485426. doi: 10.3389/fsurg.2025.1485426 (PMC11985531; doi:10.3389/fsurg.2025.1485426)
Supplement: Supplementary file 1 [file Datasheet1.pdf]

Supplementary Material 2: Top 62 Keywords with the Strongest Citation Bursts

Keywords	Year	Strength	Begin	End	1990 - 2024
**a: Treatment**					
hysterectomy	2002	2.07	**2002**	2004	
repair	2002	1.55	**2002**	2004	
rectocele repair	2007	2.25	**2007**	2008	
anterior colporrhaphy	2002	1.52	**2010**	2013	
gynecologic surgical procedures	2010	0.97	**2010**	2013	
burch colposuspension	2002	0.77	**2010**	2011	
surgery	2002	3.89	**2011**	2017	
**b: Outcome**					
distress	2001	1.54	**2001**	2003	
sexual function	2000	0.7	**2003**	2006	
injury	2008	0.8	**2008**	2011	
quality of life	2001	1.44	**2009**	2010	
**c: Anatomy & symptoms**					
bowel	2000	0.94	**2000**	2003	
vaginal vault prolapse	2000	0.83	**2000**	2001	
pelvic floor	2001	1.01	**2001**	2002	
uterine prolapse	2002	1.55	**2002**	2004	
genital prolapse	1997	1.29	**2002**	2004	
disorder	2005	1.42	**2005**	2008	
cardinal ligament	2005	0.77	**2005**	2009	
endopelvic fascia plication	2008	1.26	**2008**	2013	
levator ani	2008	1.06	**2008**	2009	
vaginal wall prolapse	2010	2.29	**2010**	2011	
damage	1996	0.8	**2011**	2013	
floor disorder	2008	2.57	**2013**	2016	
**d: Diagnosis and detection**					
prolapse staging	1996	1.6	**1996**	2001	
defecography	2000	1.17	**2000**	2001	
genuine stress incontinence	1996	0.82	**2002**	2003	
fecal incontinence	1996	0.51	**2007**	2009	
stress urinary incontinence	2000	1.04	**2017**	2018	
**e: Pathogenesis & risk factors**					
innervation	1996	1.3	**1996**	1997	
cigarette smoking	1997	0.89	**1997**	2003	
risk factor	1996	0.83	**2004**	2005	
elastic fiber homeostasis	2009	1.46	**2009**	2011	
age	2005	0.79	**2009**	2014	
delivery	2004	0.36	**2011**	2013	
epidemiology	2001	0.39	**2014**	2015	
**f: Implants**					
polypropylene mesh	2006	1.15	**2006**	2007	
**g: Research proceedings**					
randomized trial	2005	0.96	**2005**	2007	
prospective randomized trial	2007	1.68	**2007**	2008	
