# Supplementary material for: Top 100 most-cited articles on pelvic organ prolapse: a visualization and bibliometric analysis
Source: Front Surg. 2025 Mar 28;12:1485426. doi: 10.3389/fsurg.2025.1485426 (PMC11985531; doi:10.3389/fsurg.2025.1485426)
Supplement: Supplementary file 2 [file Table1.docx]

**Supplementary Material 1**. Top 100 articles with their total citations and citations per year

| **Rank** | **Article** | **Total Citations** | **Citations per year** |
| --- | --- | --- | --- |
| 1 | Bump RC, Mattiasson A, Bø K, Brubaker LP, DeLancey JO, Klarskov P, Shull BL, Smith AR. The standardization of terminology of female pelvic organ prolapse and pelvic floor dysfunction. Am J Obstet Gynecol. 1996l;175(1):10-17. | 3255 | 112.24 |
| 2 | Olsen AL, Smith VJ, Bergstrom JO, Colling JC, Clark AL. Epidemiology of surgically managed pelvic organ prolapse and urinary incontinence. Obstet Gynecol. 1997;89(4):501-506. | 2418 | 86.36 |
| 3 | Hendrix SL, Clark A, Nygaard I, Aragaki A, Barnabei V, McTiernan A. Pelvic organ prolapse in the Women's Health Initiative: gravity and gravidity. Am J Obstet Gynecol. 2002;186(6):1160-1166. | 845 | 36.74 |
| 4 | Abrams P, Andersson KE, Birder L, Brubaker L, Cardozo L, Chapple C, Cottenden A, Davila W, de Ridder D, Dmochowski R, Drake M, Dubeau C, Fry C, Hanno P, Smith JH, Herschorn S, Hosker G, Kelleher C, Koelbl H, Khoury S, Madoff R, Milsom I, Moore K, Newman D, Nitti V, Norton C, Nygaard I, Payne C, Smith A, Staskin D, Tekgul S, Thuroff J, Tubaro A, Vodusek D, Wein A, Wyndaele JJ; Members of Committees; Fourth International Consultation on Incontinence. Fourth International Consultation on Incontinence Recommendations of the International Scientific Committee: Evaluation and treatment of urinary incontinence, pelvic organ prolapse, and fecal incontinence. Neurourol Urodyn. 2010;29(1):213-240. | 737 | 49.13 |
| 5 | Wu JM, Matthews CA, Conover MM, Pate V, Jonsson Funk M. Lifetime risk of stress urinary incontinence or pelvic organ prolapse surgery. Obstet Gynecol. 2014;123(6):1201-1206. | 730 | 66.36 |
| 6 | Maher C, Feiner B, Baessler K, Schmid C. Surgical management of pelvic organ prolapse in women. Cochrane Database Syst Rev. 2013;(4):CD004014. | 635 | 52.92 |
| 7 | Jelovsek JE, Maher C, Barber MD. Pelvic organ prolapse. Lancet. 2007;369(9566):1027-1038. | 593 | 32.94 |
| 8 | Rogers RG, Coates KW, Kammerer-Doak D, Khalsa S, Qualls C. A short form of the Pelvic Organ Prolapse/Urinary Incontinence Sexual Questionnaire (PISQ-12). Int Urogynecol J Pelvic Floor Dysfunct. 2003;14(3):164-168. | 567 | 25.77 |
| 9 | Smith FJ, Holman CD, Moorin RE, Tsokos N. Lifetime risk of undergoing surgery for pelvic organ prolapse. Obstet Gynecol. 2010;116(5):1096-1100. | 559 | 37.27 |
| 10 | DeLancey JO, Morgan DM, Fenner DE, Kearney R, Guire K, Miller JM, Hussain H, Umek W, Hsu Y, Ashton-Miller JA. Comparison of levator ani muscle defects and function in women with and without pelvic organ prolapse. Obstet Gynecol. 2007;109(2 Pt 1):295-302. | 515 | 28.61 |
| 11 | Barber MD, Maher C. Epidemiology and outcome assessment of pelvic organ prolapse. Int Urogynecol J. 2013;24(11):1783-1790. | 433 | 36.08 |
| 12 | Altman D, Väyrynen T, Engh ME, Axelsen S, Falconer C; Nordic Transvaginal Mesh Group. Anterior colporrhaphy versus transvaginal mesh for pelvic-organ prolapse. N Engl J Med. 2011;364(19):1826-36. | 432 | 30.86 |
| 13 | Subak LL, Waetjen LE, van den Eeden S, Thom DH, Vittinghoff E, Brown JS. Cost of pelvic organ prolapse surgery in the United States. Obstet Gynecol. 2001;98(4):646-651. | 399 | 16.63 |
| 14 | Boyles SH, Weber AM, Meyn L. Procedures for pelvic organ prolapse in the United States, 1979-1997. Am J Obstet Gynecol. 2003;188(1):108-115. | 392 | 17.82 |
| 15 | Barber MD, Brubaker L, Nygaard I, Wheeler TL 2nd, Schaffer J, Chen Z, Spino C; Pelvic Floor Disorders Network. Defining success after surgery for pelvic organ prolapse. Obstet Gynecol. 2009;114(3):600-609. | 386 | 24.13 |
| 16 | Ellerkmann RM, Cundiff GW, Melick CF, Nihira MA, Leffler K, Bent AE. Correlation of symptoms with location and severity of pelvic organ prolapse. Am J Obstet Gynecol. 2001;185(6):1337-1338. | 385 | 16.04 |
| 17 | Nygaard I, Brubaker L, Zyczynski HM, Cundiff G, Richter H, Gantz M, Fine P, Menefee S, Ridgeway B, Visco A, Warren LK, Zhang M, Meikle S. Long-term outcomes following abdominal sacrocolpopexy for pelvic organ prolapse. JAMA. 2013;309(19):2016-2024. | 381 | 31.75 |
| 18 | Dietz HP, Simpson JM. Levator trauma is associated with pelvic organ prolapse. BJOG. 2008;115(8):979-984. | 377 | 22.18 |
| 19 | Maher C, Feiner B, Baessler K, Glazener CM. Surgical management of pelvic organ prolapse in women. Cochrane Database Syst Rev. 2010;(4):CD004014. | 318 | 21.20 |
| 20 | Shull BL, Bachofen C, Coates KW, Kuehl TJ. A transvaginal approach to repair of apical and other associated sites of pelvic organ prolapse with uterosacral ligaments. Am J Obstet Gynecol. 2000;183(6):1373-1374. | 304 | 12.16 |
| 21 | Vergeldt TF, Weemhoff M, IntHout J, Kluivers KB. Risk factors for pelvic organ prolapse and its recurrence: a systematic review. Int Urogynecol J. 2015;26(11):1559-1573. | 301 | 30.10 |
| 22 | Haylen BT, Maher CF, Barber MD, Camargo S, Dandolu V, Digesu A, Goldman HB, Huser M, Milani AL, Moran PA, Schaer GN, Withagen MI. An International Urogynecological Association (IUGA) / International Continence Society (ICS) joint report on the terminology for female pelvic organ prolapse (POP). Int Urogynecol J. 2016;27(2):165-194. | 273 | 30.33 |
| 23 | Swift SE, Tate SB, Nicholas J. Correlation of symptoms with degree of pelvic organ support in a general population of women: what is pelvic organ prolapse? Am J Obstet Gynecol. 2003;189(2):372-379 | 268 | 12.18 |
| 24 | Barber MD, Visco AG, Wyman JF, Fantl JA, Bump RC; Continence Program for Women Research Group. Sexual function in women with urinary incontinence and pelvic organ prolapse. Obstet Gynecol. 2002;99(2):281-289. | 265 | 11.52 |
| 25 | Nygaard I, Bradley C, Brandt D; Women's Health Initiative. Pelvic organ prolapse in older women: prevalence and risk factors. Obstet Gynecol. 2004;104(3):489-497. | 246 | 11.71 |
| 26 | Rogers RG, Kammerer-Doak D, Villarreal A, Coates K, Qualls C. A new instrument to measure sexual function in women with urinary incontinence or pelvic organ prolapse. Am J Obstet Gynecol. 2001;184(4):552-558. | 238 | 9.92 |
| 27 | Hall AF, Theofrastous JP, Cundiff GW, Harris RL, Hamilton LF, Swift SE, Bump RC. Interobserver and intraobserver reliability of the proposed International Continence Society, Society of Gynecologic Surgeons, and American Urogynecologic Society pelvic organ prolapse classification system. Am J Obstet Gynecol. 1996;175(6):1467-1471. | 237 | 8.17 |
| 28 | Clark AL, Gregory T, Smith VJ, Edwards R. Epidemiologic evaluation of reoperation for surgically treated pelvic organ prolapse and urinary incontinence. Am J Obstet Gynecol. 2003;189(5):1261-1267. | 229 | 10.41 |
| 29 | Weber AM, Walters MD, Piedmonte MR. Sexual function and vaginal anatomy in women before and after surgery for pelvic organ prolapse and urinary incontinence. Am J Obstet Gynecol. 2000;182(6):1610-1615. | 227 | 9.08 |
| 30 | Handa VL, Garrett E, Hendrix S, Gold E, Robbins J. Progression and remission of pelvic organ prolapse: a longitudinal study of menopausal women. Am J Obstet Gynecol. 2004;190(1):27-32. | 225 | 10.71 |
| 31 | Rortveit G, Brown JS, Thom DH, Van Den Eeden SK, Creasman JM, Subak LL. Symptomatic Pelvic Organ Prolapse: Prevalence and Risk Factors in a Population-Based, Racially Diverse Cohort. Obstetrics & Gynecology. 2007;109(6):1396-1403. | 220 | 12.22 |
| 32 | Collinet P, Belot F, Debodinance P, Ha Duc E, Lucot JP, Cosson M. Transvaginal mesh technique for pelvic organ prolapse repair: mesh exposure management and risk factors. Int Urogynecol J Pelvic Floor Dysfunct. 2006;17(4):315-320. | 190 | 10.00 |
| 33 | Maher CM, Feiner B, Baessler K, Glazener CM. Surgical management of pelvic organ prolapse in women: the updated summary version Cochrane review. Int Urogynecol J. 2011;22(11):1445-1457. | 189 | 13.50 |
| 34 | Maher C, Baessler K, Glazener CM, Adams EJ, Hagen S. Surgical management of pelvic organ prolapse in women: a short version Cochrane review. Neurourol Urodyn. 2008;27(1):3-12. | 188 | 11.06 |
| 35 | Dietz HP, Haylen BT, Broome J. Ultrasound in the quantification of female pelvic organ prolapse. Ultrasound Obstet Gynecol. 2001;18(5):511-514. | 187 | 7.79 |
| 36 | Barber MD, Visco AG, Weidner AC, Amundsen CL, Bump RC. Bilateral uterosacral ligament vaginal vault suspension with site-specific endopelvic fascia defect repair for treatment of pelvic organ prolapse. Am J Obstet Gynecol. 2000;183(6):1402-10; discussion 1402-1411. | 187 | 7.48 |
| 37 | Gyhagen M, Bullarbo M, Nielsen TF, Milsom I. Prevalence and risk factors for pelvic organ prolapse 20 years after childbirth: a national cohort study in singleton primiparae after vaginal or caesarean delivery. BJOG. 2013;120(2):152-160. | 186 | 15.50 |
| 38 | Hagen S, Stark D. Conservative prevention and management of pelvic organ prolapse in women. Cochrane Database Syst Rev. 2011;(12):CD003882. | 185 | 13.21 |
| 39 | Walker GJ, Gunasekera P. Pelvic organ prolapse and incontinence in developing countries: review of prevalence and risk factors. Int Urogynecol J. 2011;22(2):127-135. | 184 | 13.14 |
| 40 | Brown JS, Waetjen LE, Subak LL, Thom DH, Van den Eeden S, Vittinghoff E. Pelvic organ prolapse surgery in the United States, 1997. Am J Obstet Gynecol. 2002;186(4):712-716. | 184 | 8.00 |
| 41 | Abramowitch SD, Feola A, Jallah Z, Moalli PA. Tissue mechanics, animal models, and pelvic organ prolapse: a review. Eur J Obstet Gynecol Reprod Biol. 2009;144 Suppl 1:S146-S158. | 179 | 11.19 |
| 42 | Clemons JL, Aguilar VC, Tillinghast TA, Jackson ND, Myers DL. Patient satisfaction and changes in prolapse and urinary symptoms in women who were fitted successfully with a pessary for pelvic organ prolapse. Am J Obstet Gynecol. 2004;190(4):1025-1029. | 179 | 8.52 |
| 43 | Slieker-ten Hove MCP, Pool-Goudzwaard AL, Eijkemans MJC, Steegers-Theunissen RPM, Burger CW, Vierhout ME. The prevalence of pelvic organ prolapse symptoms and signs and their relation with bladder and bowel disorders in a general female population. International Urogynecology Journal. 2009;20(9):1037-1045. | 175 | 10.94 |
| 44 | Weber AM, Richter HE. Pelvic organ prolapse. Obstet Gynecol. 2005;106(3):615-634. | 175 | 8.75 |
| 45 | Rogers GR, Villarreal A, Kammerer-Doak D, Qualls C. Sexual function in women with and without urinary incontinence and/or pelvic organ prolapse. Int Urogynecol J Pelvic Floor Dysfunct. 2001;12(6):361-365. | 175 | 7.29 |
| 46 | Kerkhof MH, Hendriks L, Brölmann HA. Changes in connective tissue in patients with pelvic organ prolapse--a review of the current literature. Int Urogynecol J Pelvic Floor Dysfunct. 2009;20(4):461-474. | 173 | 10.81 |
| 47 | Kelvin FM, Maglinte DD, Hale DS, Benson JT. Female pelvic organ prolapse: a comparison of triphasic dynamic MR imaging and triphasic fluoroscopic cystocolpoproctography. AJR Am J Roentgenol. 2000;174(1):81-88. | 171 | 6.84 |
| 48 | Abrams P, Andersson KE, Apostolidis A, Birder L, Bliss D, Brubaker L, Cardozo L, Castro-Diaz D, O'Connell PR, Cottenden A, Cotterill N, de Ridder D, Dmochowski R, Dumoulin C, Fader M, Fry C, Goldman H, Hanno P, Homma Y, Khullar V, Maher C, Milsom I, Newman D, Nijman RJM, Rademakers K, Robinson D, Rosier P, Rovner E, Salvatore S, Takeda M, Wagg A, Wagner T, Wein A; members of the committees. 6th International Consultation on Incontinence. Recommendations of the International Scientific Committee: EVALUATION AND TREATMENT OF URINARY INCONTINENCE, PELVIC ORGAN PROLAPSE AND FAECAL INCONTINENCE. Neurourol Urodyn. 2018;37(7):2271-2272. | 167 | 23.86 |
| 49 | Handa VL, Harvey L, Cundiff GW, Siddique SA, Kjerulff KH. Sexual function among women with urinary incontinence and pelvic organ prolapse. Am J Obstet Gynecol. 2004;191(3):751-756. | 165 | 7.86 |
| 50 | Fialkow MF, Newton KM, Lentz GM, Weiss NS. Lifetime risk of surgical management for pelvic organ prolapse or urinary incontinence. Int Urogynecol J Pelvic Floor Dysfunct. 2008;19(3):437-440. | 163 | 9.59 |
| 51 | Handa VL, Harris TA, Ostergard DR. Protecting the pelvic floor: obstetric management to prevent incontinence and pelvic organ prolapse. Obstet Gynecol. 1996;88(3):470-478. | 161 | 5.55 |
| 52 | Denman MA, Gregory WT, Boyles SH, Smith V, Edwards SR, Clark AL. Reoperation 10 years after surgically managed pelvic organ prolapse and urinary incontinence. Am J Obstet Gynecol. 2008;198(5):555.e1-555.e5555.. | 156 | 9.18 |
| 53 | Delancey JO, Hurd WW. Size of the urogenital hiatus in the levator ani muscles in normal women and women with pelvic organ prolapse. Obstet Gynecol. 1998;91(3):364-368. | 156 | 5.78 |
| 54 | Fernando RJ, Thakar R, Sultan AH, Shah SM, Jones PW. Effect of vaginal pessaries on symptoms associated with pelvic organ prolapse. Obstet Gynecol. 2006;108(1):93-99. | 154 | 8.11 |
| 55 | Braekken IH, Majida M, Engh ME, Bø K. Can pelvic floor muscle training reverse pelvic organ prolapse and reduce prolapse symptoms? An assessor-blinded, randomized, controlled trial. Am J Obstet Gynecol. 2010;203(2):170.e1-170.e1707. | 153 | 10.20 |
| 56 | Meriwether KV, Antosh DD, Olivera CK, Kim-Fine S, Balk EM, Murphy M, Grimes CL, Sleemi A, Singh R, Dieter AA, Crisp CC, Rahn DD. Uterine preservation vs hysterectomy in pelvic organ prolapse surgery: a systematic review with meta-analysis and clinical practice guidelines. Am J Obstet Gynecol. 2018;219(2):129-146.e2. | 146 | 20.86 |
| 57 | Hagen S, Stark D, Glazener C, Dickson S, Barry S, Elders A, Frawley H, Galea MP, Logan J, McDonald A, McPherson G, Moore KH, Norrie J, Walker A, Wilson D. Individualised pelvic floor muscle training in women with pelvic organ prolapse (POPPY): a multicentre randomised controlled trial. The Lancet. 2014;383(9919):796-806. | 145 | 13.18 |
| 58 | Chapple CR, Cruz F, Deffieux X, Milani AL, Arlandis S, Artibani W, Bauer RM, Burkhard F, Cardozo L, Castro-Diaz D, Cornu JN, Deprest J, Gunnemann A, Gyhagen M, Heesakkers J, Koelbl H, MacNeil S, Naumann G, Roovers JWR, Salvatore S, Sievert KD, Tarcan T, Van der Aa F, Montorsi F, Wirth M, Abdel-Fattah M. Consensus Statement of the European Urology Association and the European Urogynaecological Association on the Use of Implanted Materials for Treating Pelvic Organ Prolapse and Stress Urinary Incontinence. Eur Urol. 2017;72(3):424-431. | 143 | 17.88 |
| 59 | Drewes PG, Yanagisawa H, Starcher B, Hornstra I, Csiszar K, Marinis SI, Keller P, Word RA. Pelvic organ prolapse in fibulin-5 knockout mice: pregnancy-induced changes in elastic fiber homeostasis in mouse vagina. Am J Pathol. 2007;170(2):578-589. | 143 | 7.94 |
| 60 | Bump RC. Racial comparisons and contrasts in urinary incontinence and pelvic organ prolapse. Obstet Gynecol. 1993;81(3):421-425. | 141 | 6.71 |
| 61 | Burrows LJ, Meyn LA, Walters MD, Weber AM. Pelvic symptoms in women with pelvic organ prolapse. Obstet Gynecol. 2004;104(5 Pt 1):982-988. | 141 | 4.41 |
| 62 | de Boer TA, Salvatore S, Cardozo L, Chapple C, Kelleher C, van Kerrebroeck P, Kirby MG, Koelbl H, Espuna-Pons M, Milsom I, Tubaro A, Wagg A, Vierhout ME. Pelvic organ prolapse and overactive bladder. Neurourol Urodyn. 2010;29(1):30-39. | 136 | 9.07 |
| 63 | Abdel-Fattah M, Ramsay I; West of Scotland Study Group. Retrospective multicentre study of the new minimally invasive mesh repair devices for pelvic organ prolapse. BJOG. 2008;115(1):22-30. | 136 | 8.00 |
| 64 | Barber MD, Walters MD, Cundiff GW; PESSRI Trial Group. Responsiveness of the Pelvic Floor Distress Inventory (PFDI) and Pelvic Floor Impact Questionnaire (PFIQ) in women undergoing vaginal surgery and pessary treatment for pelvic organ prolapse. Am J Obstet Gynecol. 2006;194(5):1492-1498. | 135 | 7.11 |
| 65 | Carley ME, Schaffer J. Urinary incontinence and pelvic organ prolapse in women with Marfan or Ehlers Danlos syndrome. Am J Obstet Gynecol. 2000;182(5):1021-1023. | 135 | 5.40 |
| 66 | Korbly NB, Kassis NC, Good MM, Richardson ML, Book NM, Yip S, Saguan D, Gross C, Evans J, Lopes VV, Harvie HS, Sung VW. Patient preferences for uterine preservation and hysterectomy in women with pelvic organ prolapse. Am J Obstet Gynecol. 2013;209(5):470.e1-470.e4706. | 133 | 11.08 |
| 67 | Sung VW, Rogers RG, Schaffer JI, Balk EM, Uhlig K, Lau J, Abed H, Wheeler TL 2nd, Morrill MY, Clemons JL, Rahn DD, Lukban JC, Lowenstein L, Kenton K, Young SB; Society of Gynecologic Surgeons Systematic Review Group. Graft use in transvaginal pelvic organ prolapse repair: a systematic review. Obstet Gynecol. 2008;112(5):1131-1142. | 130 | 7.65 |
| 68 | Rogers RG, Rockwood TH, Constantine ML, Thakar R, Kammerer-Doak DN, Pauls RN, Parekh M, Ridgeway B, Jha S, Pitkin J, Reid F, Sutherland SE, Lukacz ES, Domoney C, Sand P, Davila GW, Espuna Pons ME. A new measure of sexual function in women with pelvic floor disorders (PFD): the Pelvic Organ Prolapse/Incontinence Sexual Questionnaire, IUGA-Revised (PISQ-IR). Int Urogynecol J. 2013;24(7):1091-1103. | 127 | 10.58 |
| 69 | Boreham MK, Wai CY, Miller RT, Schaffer JI, Word RA. Morphometric analysis of smooth muscle in the anterior vaginal wall of women with pelvic organ prolapse. Am J Obstet Gynecol. 2002l;187(1):56-63. | 126 | 5.73 |
| 70 | Mouritsen L, Larsen JP. Symptoms, bother and POPQ in women referred with pelvic organ prolapse. Int Urogynecol J Pelvic Floor Dysfunct. 2003;14(2):122-127. | 126 | 5.48 |
| 71 | Tegerstedt G, Maehle-Schmidt M, Nyrén O, Hammarström M. Prevalence of symptomatic pelvic organ prolapse in a Swedish population. International Urogynecology Journal. 2005;16(6):497-503. | 123 | 6.15 |
| 72 | Fritel X, Varnoux N, Zins M, Breart G, Ringa V. Symptomatic pelvic organ prolapse at midlife, quality of life, and risk factors. Obstet Gynecol. 2009;113(3):609-616. | 121 | 7.56 |
| 73 | Clemons JL, Aguilar VC, Tillinghast TA, Jackson ND, Myers DL. Risk factors associated with an unsuccessful pessary fitting trial in women with pelvic organ prolapse. Am J Obstet Gynecol. 2004;190(2):345-350. | 121 | 5.76 |
| 74 | .Jackson SL, Weber AM, Hull TL, Mitchinson AR, Walters MD. Fecal incontinence in women with urinary incontinence and pelvic organ prolapse. Obstet Gynecol. 1997;89(3):423-427. | 121 | 4.32 |
| 75 | Gadonneix P, Ercoli A, Salet-Lizée D, Cotelle O, Bolner B, Van Den Akker M, Villet R. Laparoscopic sacrocolpopexy with two separate meshes along the anterior and posterior vaginal walls for multicompartment pelvic organ prolapse. J Am Assoc Gynecol Laparosc. 2004;11(1):29-35 | 120 | 5.71 |
| 76 | Serati M, Bogani G, Sorice P, Braga A, Torella M, Salvatore S, Uccella S, Cromi A, Ghezzi F. Robot-assisted sacrocolpopexy for pelvic organ prolapse: a systematic review and meta-analysis of comparative studies. Eur Urol. 2014;66(2):303-318. | 118 | 10.73 |
| 77 | Bø K. Pelvic floor muscle training in treatment of female stress urinary incontinence, pelvic organ prolapse and sexual dysfunction. World J Urol. 2012;30(4):437-443. | 115 | 8.85 |
| 78 | Singh K, Reid WM, Berger LA. Assessment and grading of pelvic organ prolapse by use of dynamic magnetic resonance imaging. Am J Obstet Gynecol. 2001;185(1):71-77. | 114 | 4.75 |
| 79 | Whitcomb EL, Rortveit G, Brown JS, Creasman JM, Thom DH, Van Den Eeden SK, Subak LL. Racial differences in pelvic organ prolapse. Obstet Gynecol. 2009;114(6):1271-1277. | 113 | 7.06 |
| 80 | Gutman RE, Ford DE, Quiroz LH, Shippey SH, Handa VL. Is there a pelvic organ prolapse threshold that predicts pelvic floor symptoms? American Journal of Obstetrics and Gynecology. 2008;199(6):683.e1-.e7. | 113 | 6.65 |
| 81 | Diez-Itza I, Aizpitarte I, Becerro A. Risk factors for the recurrence of pelvic organ prolapse after vaginal surgery: a review at 5 years after surgery. Int Urogynecol J Pelvic Floor Dysfunct. 2007;18(11):1317-1324. | 113 | 6.28 |
| 82 | Bai SW, Jeon MJ, Kim JY, Chung KA, Kim SK, Park KH. Relationship between stress urinary incontinence and pelvic organ prolapse. Int Urogynecol J Pelvic Floor Dysfunct. 2002;13(4):256-260. | 112 | 4.87 |
| 83 | Abdel-fattah M, Familusi A, Fielding S, Ford J, Bhattacharya S. Primary and repeat surgical treatment for female pelvic organ prolapse and incontinence in parous women in the UK: a register linkage study. Bmj Open. 2011;1(2). | 111 | 7.93 |
| 84 | Barber, MD. CLINICAL UPDATES Pelvic organ prolapse. (BMJ-BRITISH MEDICAL JOURNAL)BMJ. 2016;354. | 111 | 12.33 |
| 85 | NICE Guidance - Urinary incontinence and pelvic organ prolapse in women: management: © NICE (2019) Urinary incontinence and pelvic organ prolapse in women: management. BJU Int. 2019;123(5):777-803. | 110 | 18.33 |
| 86 | Budatha M, Roshanravan S, Zheng Q, Weislander C, Chapman SL, Davis EC, Starcher B, Word RA, Yanagisawa H. Extracellular matrix proteases contribute to progression of pelvic organ prolapse in mice and humans. J Clin Invest. 2011;121(5):2048-2059. | 108 | 7.71 |
| 87 | Bradley CS, Zimmerman MB, Qi Y, Nygaard IE. Natural history of pelvic organ prolapse in postmenopausal women. Obstet Gynecol. 2007;109(4):848-854. | 107 | 5.94 |
| 88 | Shah AD, Kohli N, Rajan SS, Hoyte L. The age distribution, rates, and types of surgery for pelvic organ prolapse in the USA. Int Urogynecol J Pelvic Floor Dysfunct. 2008;19(3):421-428. | 105 | 6.18 |
| 89 | Morling JR, McAllister DA, Agur W, Fischbacher CM, Glazener CM, Guerrero K, Hopkins L, Wood R. Adverse events after first, single, mesh and non-mesh surgical procedures for stress urinary incontinence and pelvic organ prolapse in Scotland, 1997-2016: a population-based cohort study. Lancet. 2017;389(10069):629-640. | 104 | 13.00 |
| 90 | Rahn DD, Ruff MD, Brown SA, Tibbals HF, Word RA. Biomechanical properties of the vaginal wall: effect of pregnancy, elastic fiber deficiency, and pelvic organ prolapse. Am J Obstet Gynecol. 2008;198(5):590.e1-590.e5906. | 104 | 6.12 |
| 91 | Sze EH, Sherard GB 3rd, Dolezal JM. Pregnancy, labor, delivery, and pelvic organ prolapse. Obstet Gynecol. 2002;100(5 Pt 1):981-986. | 104 | 4.52 |
| 92 | Leijonhufvud A, Lundholm C, Cnattingius S, Granath F, Andolf E, Altman D. Risks of stress urinary incontinence and pelvic organ prolapse surgery in relation to mode of childbirth. Am J Obstet Gynecol. 2011 Jan;204(1):70.e1-70.e707. | 103 | 7.36 |
| 93 | Altman D, Forsman M, Falconer C, Lichtenstein P. Genetic influence on stress urinary incontinence and pelvic organ prolapse. Eur Urol. 2008;54(4):918-922. | 103 | 6.06 |
| 94 | Gabriel B, Denschlag D, Göbel H, Fittkow C, Werner M, Gitsch G, Watermann D. Uterosacral ligament in postmenopausal women with or without pelvic organ prolapse. Int Urogynecol J Pelvic Floor Dysfunct. 2005;16(6):475-479. | 103 | 5.15 |
| 95 | .Lowenstein L, Gamble T, Sanses TV, van Raalte H, Carberry C, Jakus S, Kambiss S, McAchran S, Pham T, Aschkenazi S, Hoskey K, Kenton K; Fellow's Pelvic Research Network. Sexual function is related to body image perception in women with pelvic organ prolapse. J Sex Med. 2009;6(8):2286-2291. | 102 | 6.38 |
| 96 | Vakili B, Zheng YT, Loesch H, Echols KT, Franco N, Chesson RR. Levator contraction strength and genital hiatus as risk factors for recurrent pelvic organ prolapse. Am J Obstet Gynecol. 2005;192(5):1592-1598 | 102 | 5.10 |
| 97 | Altman D, Falconer C. Perioperative morbidity using transvaginal mesh in pelvic organ prolapse repair. Obstet Gynecol. 2007;109(2 Pt 1):303-308. | 101 | 5.61 |
| 98 | Borstad E, Abdelnoor M, Staff AC, Kulseng-Hanssen S. Surgical strategies for women with pelvic organ prolapse and urinary stress incontinence. Int Urogynecol J. 2010;21(2):179-186. | 100 | 6.67 |
| 99 | Swift SE, Pound T, Dias JK. Case–Control Study of Etiologic Factors in the Development of Severe Pelvic Organ Prolapse. International Urogynecology Journal. 2001;12(3):187-192. | 99 | 4.13 |
| 100 | Glazener C, Elders A, MacArthur C, Lancashire RJ, Herbison P, Hagen S, Dean N, Bain C, Toozs-Hobson P, Richardson K, McDonald A, McPherson G, Wilson D; ProLong Study Group. Childbirth and prolapse: long-term associations with the symptoms and objective measurement of pelvic organ prolapse. BJOG. 2013;120(2):161-168. | 98 | 8.17 |
